# Brain activity changes with emotional words in different stages of psychosis

**DOI:** 10.1192/j.eurpsy.2022.2321

**Published:** 2022-10-04

**Authors:** Pau Soldevila-Matías, Gracián García-Martí, Inmaculada Fuentes-Durá, Juan Carlos Ruiz, Laura González-Navarro, Carlos González-Vivas, Joaquim Radua, Julio Sanjuán

**Affiliations:** 1 Department of Basic Psychology, Faculty of Psychology, University of Valencia, Valencia, Spain; 2 Research Institute of Clinic University Hospital of Valencia (INCLIVA), Valencia, Spain; 3 Department of Psychology, Faculty of Health Sciences, European University of Valencia, Spain; 4 CIBERSAM, Biomedical Research Network on Mental Health Area, Madrid, Spain; 5 Biomedical Engineering Unit/Radiology Department, Quirónsalud Hospital, Valencia, Spain; 6 Department of Personality, Evaluation and Psychological Treatment, Faculty of Psychology, University of Valencia, Valencia, Spain; 7 Department of Behavioural Sciences Methodology, Faculty of Psychology, University of Valencia, Valencia, Spain; 8 Faculty of Biology, University of Barcelona, Barcelona, Spain; 9 Institut d’Investigacions Biomèdiques August Pi i Sunyer (IDIBAPS), Barcelona, Spain; 10 Centre for Psychiatric Research and Education, Department of Clinical Neuroscience, Karolinska Institutet, Stockholm, Sweden; 11 Department of Psychosis Studies, Institute of Psychiatry, Psychology and Neuroscience, King’s College London, London, United Kingdom; 12 Department of Psychiatry, University of Valencia School of Medicine, Valencia, Spain

**Keywords:** Auditory paradigm, chronic psychosis patients, emotional design model, FEP patients, Neuroimaging fMRI

## Abstract

**Background:**

To date, a large number of functional magnetic resonance imaging (fMRI) studies have been conducted on psychosis. However, little is known about changes in brain functioning in psychotic patients using an emotional auditory paradigm at different stages of the disease. Such knowledge is important for advancing our understanding of the disorder and thus creating more targeted interventions. This study aimed to investigate whether individuals with first-episode psychosis (FEP) and chronic schizophrenia show abnormal brain responses to emotional auditory processing and to compare the responses between FEP and chronic schizophrenia.

**Methods:**

Patients with FEP (n = 31) or chronic schizophrenia (n = 23) and healthy controls (HCs, n = 31) underwent an fMRI scan while presented with both emotional and nonemotional words.

**Results:**

Using HC as a reference, patients with FEP showed decreased right temporal activation, while patients with chronic schizophrenia showed increased bilateral temporal activation. When comparing the patient groups, individuals with FEP showed lower frontal lobe activation.

**Conclusion:**

To the best of our knowledge, this is the first study with an emotional auditory paradigm used in psychotic patients at different stages of the disease. Our results suggested that the temporal lobe might be a key issue in the physiopathology of psychosis, although abnormal activation could also be derived from a connectivity problem. There is lower activation in the early stage and evolution to greater activation when patients become chronic. This study highlights the relevance of using emotional paradigms to better understand brain activation at different stages of psychosis.

## Introduction

Psychosis is a major social burden due to the tendency to chronicity, the impact on physical health, and the reduction of life expectancy by a mean of 14.5 years [1–3]. A crucial issue is to understand the mechanisms involved over the course of the illness [4]. One of the main instruments used in research for first-episode psychosis (FEP) is magnetic resonance imaging (MRI) [2]. Many studies have used MRI scans to obtain information about the main volumetric changes in the brain [3]. However, the structural changes are not specific and cannot be used as predictors in individual cases, so more sensitive instruments are needed [4, 5].

Another way to study the brain is with functional MRI (fMRI), which could provide an important means of understanding what happens in the brain during the processing of different stimuli. In a previous systematic review about this issue, we showed great heterogeneity among different studies [6]. Most studies used resting states or different paradigms with different sensory modalities [7, 8], in particular, visual sensory modalities [9, 10], through the recognition of facial emotions [11, 12] and cognitive paradigms [13, 14]. However, relatively few of them have used emotional processing with the auditory modality, despite the importance of language in human emotions.

Our group developed an emotional auditory paradigm based on some of the most common symptoms reported by FEP patients [15, 16]. Under our emotional auditory paradigm, an important role of the amygdala in emotional processing in chronic patients with hallucinations can be observed [15], and some remarkable results have been found using this method in chronic samples [17–19], but the issue of brain activation changes in FEP remains unclear. Thus, we hypothesize that psychotic patients will probably show abnormal fMRI-measured activation during our emotional auditory paradigm and that these patterns may be different between patients with FEP and chronic schizophrenia. The purpose of this study is to assess for the first time the fMRI-measured brain activation in response to an emotional auditory paradigm in FEP and chronic patients (compared to a healthy control [HC] group).

## Materials and Methods

### Subject recruitment and assessment

Patients were recruited from the outpatient psychiatry clinics. People who experienced psychotic symptoms for the first time in life were submitted to the Valencia Clinic Hospital First-Episode Unit for evaluation and treatment. Patients diagnosed with schizophrenia and persistent hallucinations (according to the International Statistical Classification of Diseases and Related Health Problems, Tenth Revision [ICD-10]) were recruited from the Valencia Clinic Hospital and considered chronic patients. All patients and control participants gave written informed consent to participate in this research, which was approved by the local ethics committee. All subjects, patients, and controls were older than 18 years old.

The inclusion criteria for the patients were as follows: (a) first-episode samples, individuals who had been diagnosed with a psychotic disorder for the first time in their lives within the first 18 months after symptom onset; (b) chronic patient samples, a diagnosis of chronic auditory hallucinations that met the selection criteria for persistent hallucinations [22]; (c) the ability to speak Spanish fluently; and (d) informed consent for the study signed by the patient.

The exclusion criteria for the patients were as follows: (a) individuals who had previous contact with mental health services for psychosis, (b) evidence of psychotic symptoms precipitated by an organic cause, (c) transient psychotic symptoms resulting from acute intoxication, (d) substance abuse (except tobacco), (e) a metal prosthesis, or (f) claustrophobia. Two weeks after their diagnosis, all of the patients underwent a semistructured interview including sociological information and clinical scales. After the clinical evaluation, they were subjected to an fMRI scan, with a minimum interval of 6 months after their diagnosis.

A sample of HCs matched for age, sex, education level, and work status was recruited from the same geographic areas as the patients. The inclusion criteria for the HCs were the same as those for the patients, except for past or present psychotic symptoms. The exclusion criteria were the same as those for the patients but also included (a) past or present psychotic symptoms or past criteria for psychoactive drug abuse or dependence (except for tobacco) and (b) having a first-degree relative with a history of a diagnosed psychotic disorder. No individual in either group suffered from hearing loss.

The patients’ sociodemographic data are shown in Table 1. There were significant differences in age (0.04), work status (0.032), and coexistence (0.001) between the FEP and chronic groups (0.04).

**Table 1.** Sociodemographic characteristics of subjects.

	Healthy Controls *N* = 31	FEP *N* = 31	Chronic patients *N* = 23	F/ 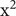	*p*-value
*Age in years: mean* (*SD*)	31.23 (7.13)	28.97 (8.77)	34.70 (8.22)	3.34	0.04*
*Sex: no.* (%)					
Male	15 (48.40)	23 (74.20)	11 (47.80)	5.47	0.065
Female	16 (51.60)	8 (25.80)	12 (52.20)		
*Educational level no.* (%)					
Elementary	1 (3.20)	3 (9.70)	1 (4.30)	4.90	0.298
Secondary	13 (41.90)	17 (54.80)	15 (65.20)		
University	17 (54.80)	11 (35.50)	7 (30.40)		
*Work status no.* (%)					
Active	13 (41.90)	11 (35.50)	7 (30.40)	16.79	0.032
Unemployed (with subsidy)	0 (0.00)	3 (9.70)	3 (13.00)		
Unemployed (without subsidy)	0 (0.00)	6 (19.40)	6 (26.10)		
Disabled	0 (0.00)	1 (3.20)	1 (4.30)		
Student	18 (58.10)	10 (32.30)	6 (21.10)		
*Coexistence no.* (%)					
Alone	16 (51.60)	1 (3.20)	0 (0.00)	37.62	0.001
Origin family	8 (25.80)	23 (74.20)	17 (73.90)		
Own family	6 (19.40)	4 (12.90)	4 (17.40)		
Descendants	1 (3.20)	0 (0.00)	0 (0.00)		
Other	0 (0.00)	3 (9.70)	2 (8.70)		

Abbreviation: FEP, first-episode psychosis.

* Tukey HSD (*p* < 0.05): Control ≠ Chronic patients; FEP ≠ Chronic patients.

The final sample consisted of 85 subjects, which included 31 FEP patients with nonaffective psychotic disorders (a manic episode with psychotic features, schizoaffective disorder, transient psychotic disorders, persistent delusional disorder, bipolar affective disorder with psychotic features, and schizophreniform disorder), 23 chronic patients with schizophrenia and persistent auditory hallucinations, and 31 HCs.

### Psychopathological measurements

Patients were assessed by personnel trained in FEP evaluation. Interviews were conducted during the first episode and a few days after the start of treatment (JS). All FEP patients underwent several psychopathological measurements, including the Clinical Global Impression [20], to provide a global rating of illness severity, improvement, and response to treatment; the Global Assessment of Function [21], to provide understanding about how well FEP patients could perform everyday activities; and the PANSS [22], which is a well-established scale that has been used to assess schizophrenia symptoms objectively. The results of the clinical measurements are summarized in Table 2. No significant clinical differences were found in any of these scales between the FEP and chronic groups.

**Table 2.** Clinical characteristics of subjects.

	FEP *N* = 31	Chronic *N* = 23		
	Mean (SD)	Mean (SD)	U-Mann Whitney	*p*
*CGI*	4.00 (0.89)	4.09 (0.79)	332.00	0.650
*GAF*	58.74 (14.80)	62.43 (14.71)	296.00	0.283
*PANSS: T*	67.26 (11.51)	70.48 (12.21)	297.00	0.297
*PANSS: P*	16.42 (4.93)	16.83 (5.36)	342.50	0.804
*PANSS: N*	16.19 (3.89)	17.26 (4.50	305.00	0.365
*PANSS: PG*	34.35 (6.27)	36.35 (6.56)	295.50	0.285
*Hamilton*	6.64 (3.78)	6.65 (4.40)	278.50	0.975
*Calgary*	3.50 (2.30)	3.30 (2.56)	253.50	0.573
*Young*	5.61 (5.93)	6.40 (7.07)	277.00	0.949
*Medication*	*n* (%)	*n* (%)	χ^2^	*p*
Paliperidone	10 (32.26)	8 (34.8)	0.38	0.996
Olanzapine	11 (35.48)	7 (30.4)		
Aripiprazole	2 (6.45)	1 (4.3)		
Risperidone	5 (16.13)	4 (17.4)		
Amisulpride	1 (3.23)	1 (4.3)		
Quetiapine	2 (6.45)	2 (8.7)		
Antipsychotic combination	3 (9.68)	3 (13.0)	0.15	0.697
Anticholinergics	3 (9.68)	1 (4.3)	0.55	0.460
Benzodiazepines	23 (74.19)	18 (78.3)	0.12	0.730
Antidepressants	3 (9.68)	3 (13.0)	0.15	0.697
Mood stabilizers	3 (9.68)	3 (13.0)	0.15	0.697

Abbreviations: CGI, Clinical Global Impression; GAF, Global Assessment of Function.

### fMRI experimental paradigm

An emotional auditory fMRI stimulation paradigm designed by our group [15, 16] was employed. The emotional response paradigm was designed to replicate the emotions related to hallucinatory experiences. Eighty-two patients with schizophrenia meeting DSM-IV criteria with AH were selected to choose words of emotional content specific to their psychoses. All patients were administered the PSYRATS, and their discourses about the content of their AH were recorded on tape. The recordings underwent transcription. Hallucinations based on complex phrases or with neutral content were ruled out. A qualitative analysis of their content in five categories was conducted: negative content and imperative tone, insults, imperative tone, and exclamations related to emotional states and positive content. The final paradigm included 13 emotional Spanish words considering their frequency of presentation in the patient’s hallucinations and 13 neutral words with a similar grammar complexity and frequency of use. For the recording procedure, a professional actor from a specialized center was hired to pronounce the words. He pronounced neutral words using a neutral tone and emotional words using an emotional tone but maintained a constant voice intensity (65 dB) [23]. A set of earphones connected by a pair of air tubes to an external audio player was used. Two different fMRI acquisitions, neutral and emotional, were conducted for each subject. The neutral words were presented first using a block design approach. Four blocks of rest and four blocks of stimulation were interleaved and presented to each participant. Each block lasted 20 s. Subjects were informed before the MR acquisition about the experiment and were asked to focus their attention on the words.

### Neuroimaging acquisition and data processing

The mean time between clinical treatment and fMRI acquisition was 4 weeks (sd +/− 6). A 3-T magnet (Achieva, Philips Medical Systems, Best, The Netherlands) was used to acquire the fMRI images. A 32-channel head coil and a dynamic echo-planar imaging T2*-weighted sequence were used (repetition time = 2.000 ms; echo time = 30 ms; flip angle 90; slice thickness = 3.50 mm with no interslice gap; acquisition matrix = 128 × 128; voxel size = 1.80 × 1.80 × 3.50 mm), covering the whole brain with 40 contiguous slices. Additionally, a 3-dimensional spoiled gradient-echo pulse T1 image was also acquired for anatomic assessment (repetition time = 13.18 ms; echo time = 7.38 ms; flip angle = 8°; NEX = 1; slice thickness = 1 with no interslice gap; acquisition matrix = 256 × 256; voxel size = 0.90 × 0.90 × 1 mm; 160 slices, thickness = 1 mm). Each fMRI session (emotional and nonemotional) had a total duration of 160 s. After the acquisition, a radiologist and a computer engineer checked the images to ensure data quality. During the acquisition, the patients were under direct observation and they asked about their experiences immediately after the fMRI procedure. Data were then anonymized and transferred to a workstation for postprocessing analysis.

Functional images were realigned to correct for involuntary movement of the patient’s head during the test. Those subjects with translation movements (*x*, *y*, *z*) >1.80 mm (voxel size) were discarded, resulting in a final sample of 31 HCs, 31 FEPs, and 23 chronic patients. Afterwards, a slice timing correction to adjust for temporal delays between the first and the last slices in each fMRI dynamic was applied. Then, a normalization algorithm to standardize the brains to a referenced template (MNI350, Montreal Neurological Institute) was used. Finally, the normalized images were smoothed with a 6-mm FWHM Gaussian smoothing kernel. These analyses were conducted using the Statistical Parametric Mapping SPM software (SPM8, Wellcome Institute, London, United Kingdom) on MATLAB R2017a (The MathWorks, Natick, MA).

### Statistics

To describe the fMRI response in each group of patients (or in HCs), we used one-sample *t-test*s. To assess whether patients with FEP (or with chronic schizophrenia) showed abnormal fMRI responses, we used two-sample *t-test*s (i.e., comparing their response to the HCs). Finally, to compare the fMRI responses between FEP and chronic schizophrenia, we also used two-sample *t-test*s. We did not use ANOVAs because we were not interested in knowing if there were differences between the three groups per se; we only used the HC data to provide a reference of normality. To correct for multiple testing, the familywise error (FWE) was applied. Only uncorrected tests for exploratory purposes were applied when no clusters survived the FWE rate correction. Additionally, an extent threshold was applied to consider only those clusters with the minimum number (k) of contiguous voxels as the expected number of voxels per cluster provided by SPM. Finally, the Automatic Area Labeling [24] atlas was used to report the areas of activation.

## Results

### fMRI response to emotional stimulation

**Figure 1.**
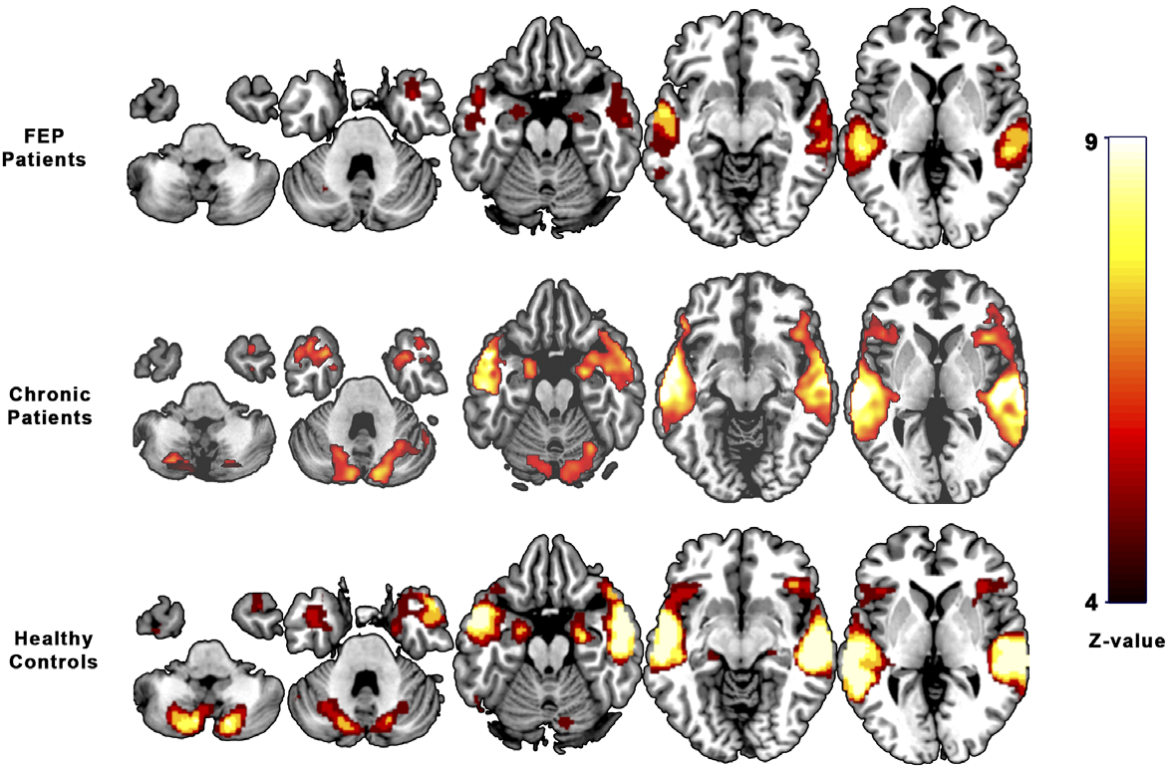
Brain activation during emotional auditory paradigm in first-episode psychosis patients, chronic psychosis patients, and healthy controls.

Patients with FEP showed activation during the emotional word task in the bilateral middle temporal (BA 21), bilateral hippocampus (left, BA 28; right, BA 35), right inferior orbitofrontal (BA 47), and left cerebellum (Table 3 and Figure 1).

**Table 3.** Regions showing activation when hearing of emotional versus neutral words in patients with FEP, healthy controls, and chronic patients.

	Peak			
	MNI	Z	*p* (FWER)	Cluster (voxels)
*Patients with FEP:*				
Left middle temporal (BA 21)	−54, −20, −4	6.34	<0.001	3,332
Right middle temporal (BA 21)	54, −34, 2	6.09	<0.001	2,754
Left hippocampus (BA 28)	−20, −4, −20	4.88	0.008	88
Right hippocampus (BA 35)	24, −10, −20	4.81	0.010	40
Right inferior orbitofrontal (BA 47)	46, 26, −2	4.78	0.011	82
Left cerebellum	−30, −62, −50	4.63	0.020	14
*Healthy controls:*				
Right middle temporal (BA 21)	56, 2, −20	7.99	<0.001	9,046
Left middle temporal (BA 21)	−56, 0, −14	7.97	<0.001	8,165
Right cerebellum	16,−76,−42	6.50	<0.001	3,032
Left inferior temporal (BA 20)	−40 −6 −50	5.82	<0.001	89
Left precentral (BA 6)	−46, 0, 54	5.26	0.002	146
Right supplementary motor area (BA 6)	8, 14, 64	5.11	0.005	289
Left cerebellum	−52, −64, −20	4.91	0.011	39
Left superior frontal (BA 32)	−12, 50, 26	4.84	0.015	43
Left putamen (BA 48)	−20, −4, 16	4.79	0.018	13
Right hippocampus (BA 20)	26, −22, −12	4.75	0.022	11
*Chronic patients:*				
Right middle temporal (BA 21)	62, −28, 2	8.83	<0.001	8,343
Left superior temporal (BA 21)	−64, −26, 4	7.83	<0.001	8,111
Right inferior frontal (BA 45)	46, 28, 18	4.79	<0.001	321
Left inferior frontal (BA 44)	−42, 10, 26	4.67	<0.001	302
Right cerebellum	14, −80, −42	4.64	<0.001	160
Left precentral (BA 06)	−46, −4, 48	4.27	0.003	138
Right precentral (BA 44)	46, 6, 30	4.25	0.004	129
Left insula (BA 47)	−36, 20, −4	4.23	0.004	97
Left cerebellum	−18 −82 −4	4.18	0.007	55
Right hippocampus (BA 20)	24, −18, −12	4.12	0.010	43
Right frontal inferior (BA 38)	50, 30, −10	4.03	0.011	31
*Patients with FEP < healthy controls*				
Right middle temporal (BA 21)	56, 6, −24	3.22	0.001	4
*Patients with FEP < chronic patients*				
Left inferior frontal (BA 44)	−44, 13, 24	4.09	0.001	67
Right inferior frontal (BA 45)	41, 22, 20	3.95	0.001	74
*Chronic patients > healthy controls*				
Left superior temporal (BA 22)	−61, −15, 5	7.83	<0.001	435
Right superior temporal (BA 48)	55, −7, −3	7.54	<0.001	574

*Note:* Additionally differences in functional activation between FEP versus healthy controls and FEP versus chronic patients were showed. Statistical threshold: familywise error rate (FWER) < 0.05 with 10-voxel cluster extent, except for the between-group comparisons (uncorrected p < 0.001).

Abbreviations: BA, Brodmann area; FEP: first-episode psychosis; FWER, familywise error rate; MNI, Montreal neurological institute.

HCs showed a similar pattern of activation, including the bilateral middle temporal (BA 21), left inferior temporal (BA 20), bilateral cerebellum, left precentral (BA 6), right supplementary motor area (BA 6), left superior frontal (BA 32), left putamen (BA 48), and right hippocampus (BA 20) (see Table 3 and Figure 1).

Chronic patients mainly activated the right middle temporal (BA 21), left superior temporal (BA 21), bilateral inferior frontal (left, BA 44; right, BA 45), bilateral cerebellum, bilateral precentral (left, BA 06; right, BA 44), left insula (BA 47), right hippocampus (BA 20), and right frontal inferior gyrus (BA 38) (see Table 3 and Figure 1).

Compared with control subjects, FEP patients did not show any differences in activation, with FWER <0.05. However, using a liberal threshold (uncorrected *p* < 0.001), they showed hypoactivation in the right temporal lobe (BA 21). Similarly, the inferior frontal gyrus (bilateral) showed a significant reduction in activation in FEP patients when compared with chronic patients (uncorrected *p* < 0.001) (BA 44, BA 45) (Table 3).

## Discussion

Three main findings resulted from the present study. First, this study provides an image comparison of FEP patients to HCs and found reduced activity in the bilateral temporal cortex (mainly on the right side). Functional studies about emotional processing in HCs have differentiated successive steps (auditory analysis and evaluative judgments) of cerebral processing involving auditory analysis within the temporal lobe [25, 26].

Second, interestingly, the CHrP group showed the opposite, greater activation in the bilateral superior temporal lobe than HCs and FEP patients during the processing of emotional words. These results suggested that the temporal lobe plays a critical but complex role in the pathogenesis of psychosis. The auditory cortex has been reported to play a pivotal role in the processing of affective prosody [26, 27].

We observed a decrease in activation in the right middle temporal lobe in the FEP group. This finding is in accordance with the assumption that this area subserves the representation of meaningful prosodic sequences in the processing of emotional words and suggests that abnormal processing of emotional words in FEP patients was conducted in the limbic system rather than in the auditory cortex [6].

Interestingly, our previous systematic review of longitudinal fMRI studies in FEP [6] showed a tendency toward normalization of brain activity after several months of antipsychotic treatment. However, in a recent study, we found different results using an emotional auditory paradigm, likely due to clinical heterogeneity and methodological issues [28].

In this sense, our results confirm that middle temporal functional alterations in FEP patients represent one key neural factor in the pathophysiology of psychosis. Considering that when HCs’ enhanced brain activity is involved, it is predominantly the temporal cortex, and passive listening to speech with prosody-caused activation relies on the right temporal cortex [29, 30].

Activation in the temporal lobes is shown (Figure 1), corresponding to the expected stimulation of auditory brain areas by the auditory paradigm used during the scan. These findings matched the images obtained from HCs. Hyperactivity in the temporal areas was observed, matching the images obtained from chronic psychotic patients in previous studies [16, 18, 19]. This established a link between enhanced activity in emotional and temporal processing regions [18, 19, 31] and their centrality in the pathophysiology of psychosis symptoms as a continuum of brain changes.

The fact that the temporal lobe and limbic regions were the most clearly affected areas in our previous studies using the same paradigm in chronic patients [19] shows how limbic system areas are implicated in the emotional response, fitting Kapur’s model in which the core of psychosis could be an abnormal “salience” to external stimuli [32] or, as more recently suggested, an abnormal connectivity between perceptual and emotional brain areas [33].

The most likely explanation for understanding the differences between our results and previous studies is that the alterations in the emotion fMRI auditory paradigm in psychotic patients are different in specific stages of the disorder and might be more noticeable during certain stages [28]. Thus, increased activation during the emotional paradigm may suggest hemodynamic dysfunctions associated with different functional changes in temporal and limbic regions [16, 18, 19] while considering psychosis as a continuum of changes depending on the illness stage and starting from mild emotional impairments to serious psychotic symptoms.

Although neuroimaging studies have revealed alterations in emotional processing [34] and social functioning [35] in FEP patients, as well as enhanced brain activity in chronic patients [16], our results provide the first evidence that activation under an emotional auditory paradigm during fMRI in FEP patients was decreased in the bilateral temporal lobes compared with HCs. This attenuation of the response to emotional auditory stimuli may be a key neurobiological mechanism associated with clinical manifestations in the early stages of psychosis. The greater activation of the temporal lobe in CHrPs might be the result of emotional memory with a key role of the amygdala and hippocampus [19].

Third, FEP patients showed lower activation in the bilateral inferior frontal area than chronic patients during the processing of emotional words. Although differences in brain activation patterns between FEP and chronic psychotic stages have been widely assessed, the results have been controversial. In a systematic review, Mwansisya et al. [30] found a pattern of frontotemporal dysfunction on fMRI in first-episode schizophrenia similar to that classically observed in chronic schizophrenia. In contrast, Li et al. [36] provided evidence for a different connectivity between FEP and chronic stages. Moreover, our group, in a recent meta-analysis, showed that the left precuneus and insula appear to be the main affected areas in FEP but not the frontal areas [14]. Nevertheless, this review included only cognitive task studies, and the differences in the results highlight the relevance of the type of paradigm used in relation to the brain activation areas.

This study has some limitations. Although most fMRI studies involve <30 patients, it is a limitation of the field, and this study must be interpreted wisely due to the relatively small sample size recruited. Differences between sample sizes in the HC and FEP groups and gender distribution should also be considered. Although the results in each clinical group were reported using the more restrictive statistical correction for multiple comparisons (FWE), the between-group test did not show any surviving cluster, and the results were presented in an exploratory way, uncorrected. Most likely, the relative number of samples in each group may cause that situation. Additional studies with more samples are needed to confirm these findings. The second limitation was that all FEP patients were on medication, mostly first-generation antipsychotics, at the time of the study, and there was no standardization of medications or doses, which could clearly affect the results. Thus, it cannot be omitted that medication dose had an impact on brain activity; however, dysfunctions in emotional processing can be unaffected by antipsychotics in FEP patients [31, 37], and their antipsychotic effects have been reported on fMRI as primarily affecting executive and memory function [38]. Moreover, dysfunction in emotional processing has been obtained previously in FEP antipsychotic-naïve patients [39]. Finally, another limitation is the difference in the mean age between the FEP and chronic groups (*p* = 0.04), which could also affect the fMRI results.

Conversely, this study has some strengths that should be noted. The main strength is that this is the first cross-sectional study investigating fMRI changes in FEP and CHrP samples with an emotional auditory paradigm. Paradigm selection is a key question in neuroimaging, and there is a lack of studies using noncognitive tasks during scans. This study provides relevant information to understand the dysfunction of the emotional response in FEP patients compared with CHrP. The study used a rigorous approach to analyze brain activity during the emotional fMRI auditory paradigm.

## Conclusions

To the best of our knowledge, this is the first study to identify fMRI activity involved in emotional auditory processing in FEP and CHrP patients. Our results suggested that the temporal lobe might be a key issue in the physiopathology of psychosis, although abnormal activation could also be derived from a connectivity problem. This role of the temporal lobe changes depending on the stage of the illness. Lower activation in the early stage might progress to greater activation when patients become chronic. This study highlights the relevance of using not only cognitive tasks but also emotional paradigms to better understand brain activation at different stages of psychosis and the potential of this method as a biomarker for the prediction of FEP outcomes.

## Data Availability

The data that support the findings of this study are not publicly available due to the privacy of research participants. Requests may be sent to the Clinical Research Ethics Committee of INCLIVA.
